# Circulating CD133+CD34+ progenitor cells inversely correlate with soluble ICAM-1 in early ischemic stroke patients

**DOI:** 10.1186/1479-5876-9-145

**Published:** 2011-08-26

**Authors:** Tanya Bogoslovsky, Maria Spatz, Aneeka Chaudhry, Dragan Maric, Marie Luby, Joseph Frank, Steven Warach

**Affiliations:** 1 Stroke Diagnostics and Therapeutics Section, National Institute of Neurological Disorders and Stroke, National Institutes of Health, 10 Center Dr, Bethesda, MD, 20892-1401, USA; 2 Stroke Branch, National Institute of Neurological Disorders and Stroke, National Institutes of Health, 10 Center Dr Bethesda, MD, 20892-1401, USA; 3 NINDS Flow Cytometry Core Facility, National Institute of Neurological Disorders and Stroke, National Institutes of Health, Bethesda, 49 Convent Dr Bethesda, MD, 20892-4479, USA; 4 Frank Laboratory, Clinical Center, National Institute of Biomedical Imaging and Bioengineering, National Institutes of Health, 10 Center Dr, Bethesda, MD, 20892-1401, USA

## Abstract

**Background and Purpose:**

Both endothelial progenitor cells (EPC) and markers of neuroinflammation are candidate biomarkers for stroke severity and outcome prediction. A relationship between EPC and neuroinflammatory markers in early stroke is not fully elucidated. The objectives were to investigate correlations between EPC and neuroinflammation markers (adhesion molecules ICAM-1, VCAM-1, E-selectin, tumor necrosis factor (TNF)-α, interleukin (IL)-6, endothelin (ET)-1, markers of tissue injury (matrix metalloproteinases (MMP)-9 and tissue inhibitor of matrix metalloproteinases (TIMP)-1) in early stroke patients.

**Methods:**

We prospectively recruited symptomatic patients with ischemic cerebrovascular disease. We assessed stroke severity by using of acute (diffusion-weighted imaging (DWI) and final lesion volumes (fluid attenuated inversion recovery (FLAIR). We measured serum soluble ICAM-1, VCAM-1, E-selectin, MMP-9, TIMP-1 and plasma TNF-α, IL-6, ET-1 by ELISA, and quantified EPC in mononuclear fraction of peripheral blood on days 1 and 3 in 17 patients (mean(SD) age 62(14), with admission National Institutes of Health Stroke Scale (NIHSS) 10(8)) selected from 175 patients with imaging confirmed ischemic stroke. Non-parametric statistics, univariate and multivariate analysis were used.

**Results:**

Only ICAM-1 inversely correlated with EPC subset CD133+CD34+ on day 1 (Spearman r = -0.6, p < 0.01) and on day 3 (r = -0.967, p < 0.001). This correlation remained significant after adjustment for age and NIHSS (beta -0.992, p < 0.004), for glucose and systolic blood pressure (beta -0.86, p < 0.005), and for white blood cells and hematocrit (beta -1.057, p < 0.0001) on day 3. MMP-9 (r = 0.509, p < 0.04) and MMP-9/TIMP-1 (r = 0.59, p < 0.013) on day 1 correlated with acute lesion volume. Both IL-6 (r = 0.624, p < 0.01) and MMP-9/TIMP-1 (r = 0.56, p < 0.02) correlated with admission NIHSS.

**Conclusion:**

Our study showed that high ICAM-1 is associated with low CD133+CD34+subset of EPC. Biomarkers of neuroinflammation may predict tissue injury and stroke severity in early ischemia.

## Background

Stroke is a devastating condition and a second leading cause of death worldwide. Usefulness of blood biomarkers for evaluation of stroke severity and outcome prediction represents an emerging field of biomedical research [1]. Numerous molecular biomarkers have been investigated, however, due to complexity of stroke pathophysiology [2,3], no single biomarker has been yet established, and new candidate biomarkers are needed.

Endothelial progenitor cells (EPC) are participants in a reparative angiogenesis [4] and may serve as markers of acute phase stroke severity [5]. Increased EPC predict favorable stroke outcome [6] and have a potential for outcome prediction of cardiovascular diseases [7,8]. EPC are activated through various angiogenic signals, such as vascular endothelial factor, angiopoietin- 2, stromal-derived factor-1α [9-11] released by hypoxic cells, and are mobilized from bone marrow after activation of matrix metalloproteinases (MMPs)[12], and then recruited to sites of vessel injury to aid endothelial recovery. After ischemic tissue injury, numerous cytokines, growth factors and chemokines (tumor necrosis factor (TNF)-alfa, interleukins (IL), adhesion molecules, endothlelins (ET)) are released locally [2,3,13]. They can contribute to neuroinflammation, and they can be subsequently detected in peripheral blood [12,13]. Biomarkers of neuroinflammation [14-18] and of tissue damage [19,20] are extensively studied as markers of stroke severity and for outcome prediction. However, the relationship of neuroinflammtory factors and severity of tissue injury to rate of neovascularization propagated by EPC in acute ischemia is currently unknown.

We hypothesized that pronounced neuroinflammation inhibits neovascularization in acute ischemia, and EPC levels are inversely related to of neuroinflammatory markers in early ischemic stroke patients. The first objective of this study was to determine correlations between EPC and major cellular and cytokine biomarkers of neuroinflammation (intercellular adhesion molecule (ICAM)-1, vascular adhesion molecule (VCAM)-1, E-selectin, TNF-α, IL-6, endothelin (ET)-1), and tissue injury/remodeling (MMP-9) in early stroke. The second objective was to evaluate relation of these biomarkers to stroke severity.

This report demonstrates that ICAM-1 is inversely correlated with CD133+CD34+ subset of EPC on both days 1 and 3 after stroke onset. Marker of tissue injury MMP-9 and pro-inflammatory cytokine IL-6 were associated with acute lesion volume and with admission National Institutes of Health Stroke Scale (NIHSS).

## Patients And Methods

### Study design

This is a prospective pilot study of consecutive patients with imaging confirmed acute stroke admitted to the single stroke center at the Washington Hospital Center (WHC) between October 2008 and May 2009, whose first MRI imaging was within 24 hr of the time of last seen normal (LSN).

### Ethical approval

The study was approved by the NIH and WHC IRB (Institutional Review Board). Written informed consent was obtained from all patients participating in the study according to the institutional guidelines of NIH and WHC.

### Study population and enrollment criteria

A group of 323 consecutive patients referred upon suspicion of an acute cerebrovascular event. After accurate evaluation, forty (12%) of these patients were diagnosed with hemorrhagic stroke, 13 (4%) with TIA, 96 (30%) with stroke mimics (non-ischemic ethiology), and 175 (54%) had imaging confirmed ischemic stroke. Forty-one (23%) of the admitted ischemic stroke patients participated in the Natural History Protocol "Evaluation, Pathogenesis, and Treatment of Patients with or at Risk for Cerebrovascular Disease", which is a prospective observational study including patients 18 years old or older with suspected acute stroke or TIA. After signing informed consent the participants undergo serial brain MRI and blood draws. The patients were included consecutively.

Exclusion criteria were as follows: 1) contraindications to MRI, 2) latency form last seen normal (LSN) to blood draws more than 72 hours, 3) signs or symptoms of hematological, hepatic, infectious diseases, active malignancy, or known recent surgery, which may interfere with levels of the studied biomarkers, 4) peripheral vessel status that did not allow blood collection. Blood collection was performed on working hours (Monday through Friday) that led to lost of a few patients otherwise consented for the Natural History Protocol. The final study population included 17 patients (10% of all ischemic stroke patients) and did not differ significantly from the total stroke cohort admitted to the hospital regarding demographics, medication, or stroke subtypes. The modified National Institutes of Health Stroke Scale score was performed within 30 min the time of initial brain MRI.

### Brain MRI imaging and lesion volume measurements

MRI was performed using a 3T (Philips Medical Systems) clinical scanner [21]. Lesion volumes were measured from diffusion weighted image (DWI) and fluid attenuated inversion recovery (FLAIR) using a semi-automated quantitative method [22]. We used acute (DWI) and final infarct volumes (FLAIR) as surrogate markers of stroke severity. Growth of lesion volume was calculated as a difference between final FLAIR and baseline DWI lesion volumes. Baseline DWI (baseline lesion volume) was performed at 9 ± 8 hr after LSN. Day 1 DWI was performed at 37 ± 19 hr and FLAIR (final lesion volume) was performed at 10 ± 13 days after LSN.

### Sample collection

Peripheral blood was collected at 31 ± 13 hr (day 1) and 76 ± 29 hr (day 3) after LSN. Blood was collected by venipuncture in 4 Vacutainer CPT (Cell Preparation Tube, BD, Franklin Lakes, NJ) and centrifuged at 2000xg for 25 minutes. The mononuclear layer was isolated and resuspended in autologous plasma containing 10% dimethyl sulfoxide (DMSO) and stored at -80°C until EPC analysis [23]. Plasma was separated and stored at -80°C until cytokine measurements.

### Measurements of biomarkers

EPC populations were identified using combinations of surface markers (CD34+, CD133+, VEGFR2+). Briefly, 2 × 10^6 ^mononuclear cells were incubated in 120 μL buffered saline containing 2% BSA with 20 μL Fc-blocking agent (Miltenyi Biotech, Auburn, CA) for 10 minutes at 25°C to inhibit non-specific binding of antibodies. Thereafter, the cells were incubated at 4°C for 30 minutes with 20 μL CD133/AC133-PE (Miltenyi Biotech, Auburn, CA), 20 μL VEGFR2-FITC (R&D Systems, Minneapolis, MN) and 20 μL CD34-ECD (Beckman Coulter, Brea, CA) in a total volume of 200 μL. The cells were washed twice before resuspension in 400 μL of stain buffer (BD Biosciences, Franklin Lakes, NJ). Just prior to analysis on a FACS Vantage SE (BD Biosciences), DAPI nuclear dye was added to the cell suspension to allow for viability gating that was constantly 70 ± 3%, across the two collection timepoints for all patients. Finally, a minimum of 1 × 10^6 ^live cells were collected, and FACS analysis was performed in triplicate for each sample. Medians of three measurements were calculated, and the resulting EPC counts are expressed as a percentage of total mononuclear cells in each sample.

Serum total MMP-9 (minimal detectable value (MDV) 0.156 ng/mL), TIMP-1 (MDV 0.08 ng/mL), E-selectin (MDV 0.009 ng/mL), ICAM-1 (MDV 0.096 ng/mL), VCAM-1 (MDV 0.6 ng/mL) and plasma IL-6 (MDV 0.7 pg/mL), and ET-1 (MDV 1.0 pg/mL) were measured by commercially available sandwich enzyme-linked immunoassays (ELISA) (Quantikine, R&D Systems Inc Minneapolis, MN) in duplicate samples according to the manufacturer instructions. TNF-α was measured in duplicate by ultrasensitive ELISA (ALPCO, NH), (MDV 0.5 pg/mL). MMP-9/TIMP-1 ratios were calculated.

### Statistical analysis

Descriptive and frequency analysis was obtained for all data. Because biomarkers were not normally distributed, data are presented as median with range. Correlations were made using the two-tailed Spearman rank test. Differences between groups were assessed either by the two-tailed Mann-Whitney test or the Kruskall-Wallis test. Differences between repeated measurements were calculated by the two-tailed Wilcoxon rank test. Linear regression was used for adjustments for major risk stroke factors and factors potentially influencing biomarkers' and EPC levels. SPSS software version 16.0 was used. The level of significance was set up at p < 0.05.

## Results

### Patients' characteristics

In total, 17 patients from the total cohort of stroke patients met the inclusion criteria (10% of all ischemic stroke patients). The study population did not differ significantly from the total stroke cohort admitted to the hospital in respect to demographics or medication (Table 1). Accordingly to the TOAST criteria the stroke types were diagnosed as follows: three patients (18%) had large artery atherosclerosis, three (18%) had small artery occlusion, seven (41%) had cardioembolic stroke, three (18%) had other determined causes, and one patient (6%) had an undetermined cause of stroke. Hence, the percentage of cardioembolic strokes and the percentage of patients who received rTPA or endovascular treatment were higher in the study group. Baseline DWI lesion volume was 17 (3-42) (medians, (first and third quartile). Day 1 DWI lesion volume was 20 (5-46) ml, FLAIR was 27 (4-76) ml and lesion growth volume was 9 (1-39) ml.

**Table 1:** Demographics, vascular risk factors, stroke subtypes, biochemical and clinical data, admission medication and treatment of the study population

*Demographics*	
Age, yr	62 ± 14
Gender (% female)	9 (53%)
Race (% Caucasians)	4 (24%)
NIHSS on admission	10 ± 8
Pre-admission modified Rankin Scale	1 ± 1
***Cardiovascular risk factors*** Hypertension	14 (82%)
DM	10 (59%)
Hyperlipidemia	7 (41%)
CAD	4 (24%)
AF	6 (35%)
Previous stroke	2 (12%)
Smoking	2 (12%)
ETOH	3 (18%)
Former cancer	2 (12%)
Illicit drug	1 (6%)
Previous ICH	1 (6%)
***Biochemical parameters*** WBC number (× 10^3^/µL)	8.5 ± 3.9
RBC number (×10^9^/µL)	4.56 ± 0.85
Hb, mg/dL	13.2 ± 2.7
Ht, %	39.9 ± 7.2
SBP, mmHg	153 ± 33
DBP, mmHg	88 ± 18
Glucose level, mg/dl	179 ± 107
Platelets number (×10^9^/µL)	265 ± 63
INR	1.1 ± 0.3
***Medication on admission*** Statins	5 (29%)
Aspirin	6 (35%)
Coumadin	2 (12%)
ACE blockers	4 (24%)
ARB Βeta-blockers Insulin	3 (18%) 5 (30%) 3 (18%)
Intervention/rtPA (IV)	5/3 (29%/18%)

Data are represented as mean ± SD or %.

### Temporal profiles of biomarkers in the acute stroke patients

Descriptive data on temporal profiles of biomarkers in acute patients are presented in the Table 2. E- selectin was the only one biomarker that decreased (p < 0.039, Wilcoxon signed rank test) on day 3 in the group of stroke patients compared to day 1. EPC subsets in the stroke population were quantified at day 1, as follows: 0.02% (0.01-0.04) CD133^+^CD34^+^, 0.01% (0.001-0.02) CD133^+^VEGFR2^+^, 0.002% (0.0006-0.004) CD34^+^VEGFR2^+ ^and 0.0009% (0.0003-0.0018) CD34^+^CD133^+^VEGFR2^+ ^(medians and first and third quartile). Detailed information about characteristics of EPC subsets is presented elsewhere [5].

**Table 2:** Descriptive statistics (median and range) and Wilcoxon signed rank test of the biomarkers measured on Day 1 and Day 3 in the stroke patients

	Day 1	Day 3	Z	P <
Plasma TNF-α, pg/mL	0.7 (0-12)	0.9 (0-32)	-1.836	0.07
Plasma IL-6, pg/mL	9 (0-58)	8 (0.4-100)	-0.628	0.5
Plasma ET-1, pg/mL	7 (7-18)	9 (5-17)	-0.968	0.3
Serum E-selectin, ng/mL	61 (36-77)	52 (43-65)	-2.062	0.04*
Serum ICAM-1, ng/mL	233 (14-403)	200 (18-293)	-0.157	0.9
Serum VCAM-1, ng/mL	642 (427-1125)	655 (47-1165)	-1.098	0.3
Serum MMP-9, ng/mL	398 (0-1384)	438 (292-542)	-0.178	0.8
Serum TIMP-1, ng/mL	264(195-540)	285 (153-376)	-1.067	0.3
MMP-9/TIMP-1	1.3 (0-4.8)	1.4 (0.2-5.8)	-0.267	0.8

*P < 0.05

### Correlations between biomarkers and EPC in acute stroke patients

**Figure 1:**
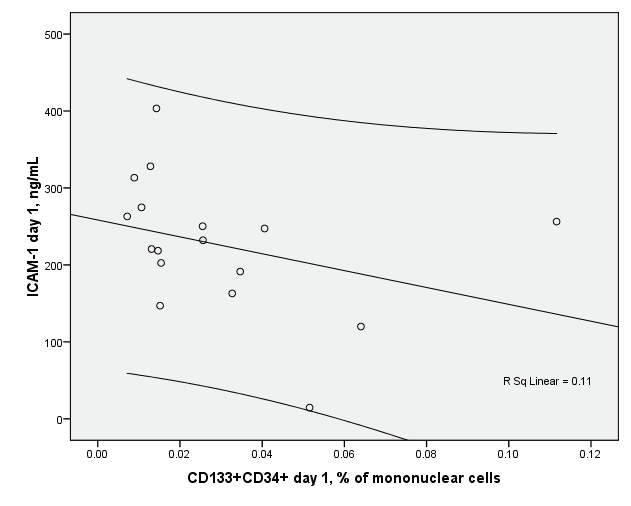
**Scatterplot of serum soluble ICAM-1 and circulating CD34+CD133+ EPC on day 1 in the cohort of stroke patients (Spearman r = -0.6, p < 0.01, n = 17, with 95% CI)**.

**Figure 2:**
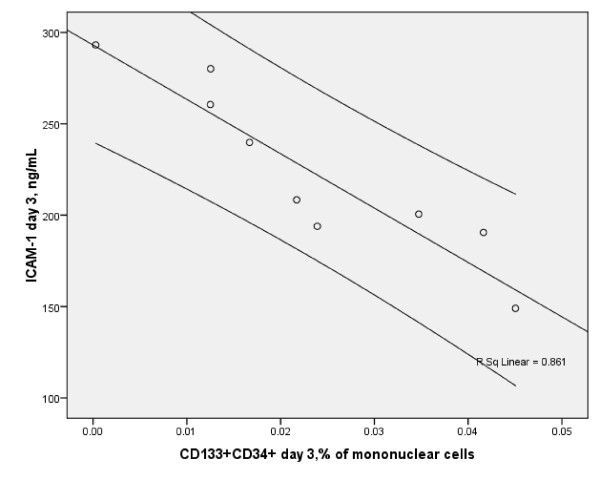
Scatterplot of serum soluble ICAM-1 and circulating CD34+CD133+ EPC on day 3 (Spearman r = -0.967, p < 0.001, n = 9 with 95% CI) in the stroke patients

CD133+CD34+ subsets inversely strongly correlated with serum ICAM-1 level on day 1 (r = -0.6, p < 0.01), (Figure 1) and on day 3 (r = -0.967, p < 0.001), (Figure 2), although, no correlations between EPC and other biomarkers were observed on both days.

On day 3, the correlations between CD133+CD34+ and ICAM-1 remained significant after adjustment for age and NIHSS (beta -0.992, p < 0.004), for glucose and systolic blood pressure (SBP) (beta -0.86, p < 0.005), and for white blood cells (WBC) and hematocrit (beta -1.057, p < 0.0001). However, these adjustments resulted in loss of correlation between CD133+CD34+ and ICAM-1 on day 1. In addition, similar models of adjustment led to loss in significance of the correlations between IL-6 and ET-1, and MMP-9, and MMP-9/TIMP-1.

### Correlations between biomarkers and lesion volume

**Figure 3:**
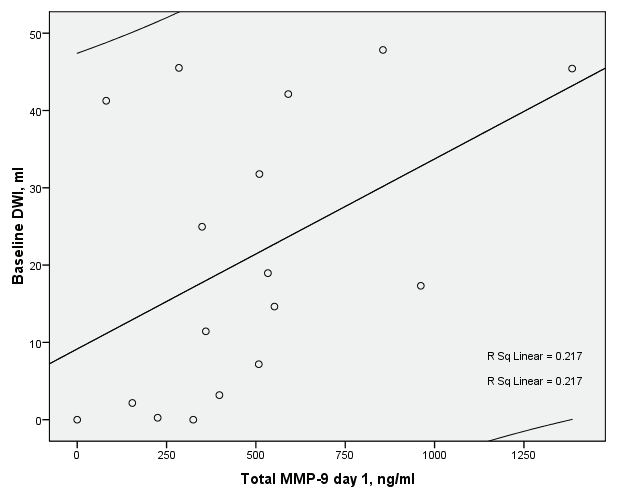
**Scatterplot of baseline lesion volume and day 1 total serum MMP-9 (Spearman r = 0.509, p < 0.037, n = 17 with 95% CI) in the stroke patients**.

**Figure 4:**
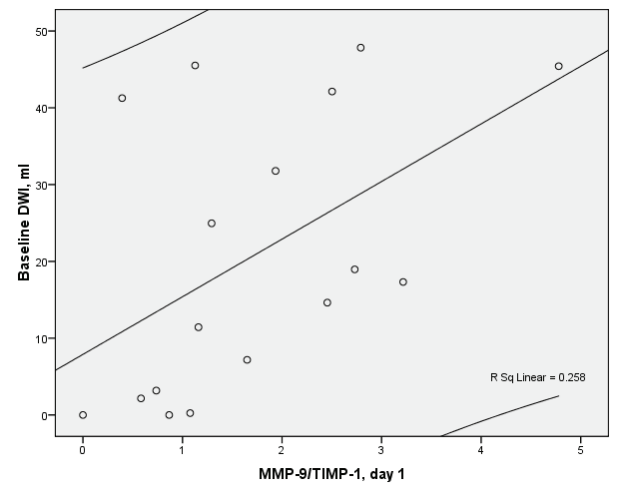
**Scatterplot of baseline lesion volume and day 1 MMP-9/TIMP-1 (Spearman (r = 0.59, p < 0.013, n = 17 with 95% CI) in the stroke patients**.

At day 1, DWI acute lesion volume strongly correlated with MMP-9 (r = 0.509, p < 0.037), (Figure 3), and MMP-9/TIMP-1(r = 0.59, p < 0.013), (Figure 4). No correlations between other biomarkers on day 1 and acute or final lesion volumes were found.

### Association of biomarkers with patients' demographics, stroke severity, risk factors and medication

Admission NIHSS strongly correlated with day 1 IL-6 (r = 0.624, p < 0.01) and with MMP-9/TIMP-1 (r = 0.523, p < 0.038). No influence of sex, race, hypertension, DM, hyperlipidemia, CAD, AF and smoking on biomarkers was found. Among risk factors, presence of previous stroke was associated with decreased levels of E-selectin (z -2.236, p = 0.015, Mann-Whitney). However, only two patients had a previous stroke, those patients had lower levels of E-selectin (median 42, range 37-47, n = 2) than the patients without previous stroke (median 63, range 51-77, n = 15). Accordingly, these data have to be interpreted with caution.

## Discussion

The major findings of our pilot study are: 1) consistent inverse correlation between circulating EPC and soluble ICAM-1, 2) correlation of acute DWI lesion volume with day 1 MMP-9 and MMP-9/TIMP-1 ratio, 3) association of admission NIHSS with day 1 IL-6 and MMP-9.

We measured soluble ICAM-1, which is easily assessable in clinical setting. It shown to be proportional to cellular ICAM-1, which is expressed on endothelial cells [24] and, therefore, soluble ICAM-1 may most likely reflect endothelial function. Up-regulation of ICAM-1 is an essential step mediating transmigration of leukocytes through perturbed endothelium and exacerbating reperfusion injury [25]. Up-regulation of endothelial expression of ICAM-1 is shown in acute ischemic brain tissue in humans [26]. Increase of ICAM-1 is linked to stroke-related neurological deterioration [27], and poor short-term stroke prognosis [15]. The most important finding in the study is the link between high ICAM-1 and low levels of circulating CD133+CD34+ EPC in early stroke. EPC represent a cumulative index of cerebrovascular function [7], and are decreased in severe atherosclerosis [8], in patients with increased cardiovascular risk [7], and in severe strokes [5]. The precise mechanism of this association remains to be determined; however, a receptor ICAM-1/CD18 is shown to be expressed on EPC and plays an essential role in their recruitment [28]. Importantly, this subset CD133^+^CD34^+ ^represents immature population of strongly proliferating progenitor cells, and is co-expressed in hematopoietic progenitors [29]. Previous experimental studies showed a plateau in expression of ICAM-1 by endothelial cells after TNF-α activation between days 1-3 [24], and after the stroke onset a persistency in soluble ICAM-1 levels on days 1, 3 and 14 [17] was detected; our follow-up data on day 3 is in line with these findings. The main impact of our study is an attempt to identify factors, which contribute to the impaired EPC mobilization in stroke. A recent study showed an association between high ET-1 levels and low EPC mobilization after acute myocardial infarction [30]. Temporal profiles of EPC in acute stroke and AMI share similar patterns [6,31]. Both ET-1 and ICAM-1 are considered to be important markers of endothelial dysfunction. We failed to show a correlation between low EPC and high ET-1 in our stroke cohort. However, our study in own way supports the reported observation [30] emphasizing that endothelial dysfunction can be related to impaired neovascularization carried out by EPC.

In our stroke cohort, day 1 MMP-9 and MMP-9/TIMP-1 ratio correlated with acute DWI lesion volumes. Acute lesion volumes are linked with clinical stroke severity [32] and widely used for outcome prediction. Our findings are in accordance with numerous clinical studies showing associations of MMP-9, responsible for degradation of basal lamina and extracellular matrix components, with increased hemorrhagic transformation in acute stroke [33], BBB disruption [20] and worse outcomes [19].

Increased levels of pro-inflammatory IL-6 are correlated with acute infarct volume measured on computed tomography (CT) and with stroke outcome measured by modified Rankin scale (mRs) at 3 months [18]. Increase of IL-6 is associated with early neurological worsening [34], and with 3 month poor outcome (mRs 3-5, death or dependency)[35]. Our data is in line with previous findings, although our stroke cohort did not include many severe stroke patients.

Focal ischemia initiates an inflammatory response, which amplifies growth of ischemic lesion in acute phase of stroke [2,3,14]. The detected high ICAM-1 level, an important marker of neuroinflammation, is associated with low levels of EPC in early stroke, which supports our hypothesis that early inflammation inhibits neovascularization. The findings also suggest that combination of both markers may be useful for stroke outcome prediction in clinical setting. However, a larger prospective study is needed to confirm our findings. The major limitation of the study is a small sample size. On the other hand, we carefully selected patient population excluding any indication of underlying inflammatory condition. Other limitations of the study include absence of the age- and sex- matched controls.

## Conclusion

High levels of ICAM-1 are linked to low levels of CD133+CD34+ subset of EPC. Our findings also support the hypothesis that biomarkers of neuroinflammation and remodeling are related to severity of tissue injury in early ischemia.

## List of Abbreviations

EPC: endothelial progenitor cells; DWI: diffusion-weighted imaging; FLAIR: fluid attenuated inversion recovery; ICAM: intercellular adhesion molecule; VCAM: vascular adhesion molecule; TNF: tumor necrosis factor; MMP: matrix metalloproteinase; TIMP: tissue inhibitor of matrix metalloproteinase; ET: endothelin; IL: interleukin; NIHSS: National Institutes of Health Stroke Scale; WBC: white blood cells; RBC: red blood cells; Hb: hemoglobin; Ht: hematocrit; INR: International Normalized Ratio; SBP: systolic blood pressure; DBP: diastolic blood pressure; ACE: angiotensin convertase enzyme; ARB: angiotensin receptor blockers; rtPA: recombinant tissue plasminogen activator; ETOH: chronic alcohol consumption.

## Competing interests

The authors declare that they have no competing interests.

## Authors' contributions

TB carried out the ELISA measurements, participated in the design of the study and performed the statistical analysis and drafted the manuscript. DM carried out the flow cytometry. AC carried preparation of EPC for enumeration. ML carried out volume measurements. JF and MS participated in the design of the study and in the statistical analysis. SW conceived of the study, and participated in its design and coordination. All authors read and approved the final manuscript.
